# Adaptive spatial-temporal neural network for ADHD identification using functional fMRI

**DOI:** 10.3389/fnins.2024.1394234

**Published:** 2024-05-30

**Authors:** Bo Qiu, Qianqian Wang, Xizhi Li, Wenyang Li, Wei Shao, Mingliang Wang

**Affiliations:** ^1^School of Computer Science, Nanjing University of Information Science and Technology, Nanjing, China; ^2^Department of Radiology and BRIC, University of North Carolina at Chapel Hill, Chapel Hill, NC, United States; ^3^College of Computer Science and Technology, Nanjing University of Aeronautics and Astronautics, Nanjing, China; ^4^Nanjing Xinda Institute of Safety and Emergency Management, Nanjing, China

**Keywords:** dynamic functional connectivity, temporal dependency, local and global evolution patterns, adaptive learning, fMRI

## Abstract

Computer aided diagnosis methods play an important role in Attention Deficit Hyperactivity Disorder (ADHD) identification. Dynamic functional connectivity (dFC) analysis has been widely used for ADHD diagnosis based on resting-state functional magnetic resonance imaging (rs-fMRI), which can help capture abnormalities of brain activity. However, most existing dFC-based methods only focus on dependencies between two adjacent timestamps, ignoring global dynamic evolution patterns. Furthermore, the majority of these methods fail to adaptively learn dFCs. In this paper, we propose an adaptive spatial-temporal neural network (ASTNet) comprising three modules for ADHD identification based on rs-fMRI time series. Specifically, we first partition rs-fMRI time series into multiple segments using non-overlapping sliding windows. Then, adaptive functional connectivity generation (AFCG) is used to model spatial relationships among regions-of-interest (ROIs) with adaptive dFCs as input. Finally, we employ a temporal dependency mining (TDM) module which combines local and global branches to capture global temporal dependencies from the spatially-dependent pattern sequences. Experimental results on the ADHD-200 dataset demonstrate the superiority of the proposed ASTNet over competing approaches in automated ADHD classification.

## 1 Introduction

Attention Deficit Hyperactivity Disorder (ADHD), with an incidence rate of 7.2% (Thomas et al., 2015), has been the most prevalent psychiatric disorder among adolescents. Individuals affected by ADHD often commonly encounter difficulties in behavior management, hyperactivity, and maintaining attention or focus. However, due to complex pathological mechanisms of ADHD, most current diagnosis methods for ADHD primarily rely on clinical behavioral observations, which may be subjective. Undoubtedly, computer aided diagnosis methods provides a more objective and comprehensive assessment, aiming to help enhance accuracy and efficiency of ADHD diagnosis.

Resting-state functional magnetic resonance imaging (rs-fMRI), which can capture changes in blood flow in response to stimulation, has emerged as a valuable tool for diagnosing diverse psychiatric diseases (Damoiseaux, 2012; Jie et al., 2014a; Wang et al., 2019a; Wang M. et al., 2022). Functional connectivities (FCs), derived from the rs-fMRI, provide insights into quantifying the temporal correlation of functional activation across different brain regions. FCs are usually defined by the correlation (i.e., Pearson correlation) between blood-oxygen-level-dependent (BOLD) signals. In recent years, researchers have designed various learning-based computer-aided diagnostic methods for ADHD analysis and they have observed that ADHD patients exhibit abnormal FCs between ROIs. These abnormal FCs can serve as potential biomarkers for clinical diagnosis of ADHD. Previous FC-based methods were usually conducted with the assumption that FC remains constant during fMRI recording. Recently, more and more studies have confirmed that brain activity is actually dynamic (Arieli et al., 1996; Makeig et al., 2004; Onton et al., 2006), and analysis based on this can reveal changes in FCs over time (Du et al., 2018; Zhang et al., 2021). These changes can help us understand how cognitive states evolve over time, which is critical for better understanding the pathology of brain diseases. For this reason, there has been a shift toward dynamic connectivity analysis in recent efforts (Bahrami et al., 2021; Wang Z. et al., 2022; Yang et al., 2022).

Specifically, most dFC-based methods can be roughly categorized into two groups: (1) conventional machine learning methods (Wang et al., 2017; Vergara et al., 2018; Feng et al., 2022) and (2) deep learning methods (Wang et al., 2019b; Yan et al., 2019; Lin et al., 2022). Previous machine learning methods first extract features manually and then feed them into subsequent prediction models. These approaches take fMRI feature learning and downstream model training as independent processes, possibly leading to suboptimal model performance. In contrast, deep learning methods usually perform feature learning and downstream prediction tasks in an end-to-end manner, which can learn task-oriented discriminative fetatures to facilitate ADHD identification. By automatically learning features from the dFC network, deep learning methods provide a cohesive framework for feature learning and classification. Considering that the functional connectivity network can be mathematically modeled as a graph, graph convolutional network (GCN), renowned for their effectiveness in processing the graph data, has been widely used in FC analysis. However, it is worth noting that many GCN methods are designed based on predefined FCs, which hinders the adaptive learning of interactions between different brain ROIs. Furthermore, most dFC-based methods only focus on temporal dependencies between adjacent timestamps, ignoring important global dynamic evolution.

**Figure 1:**
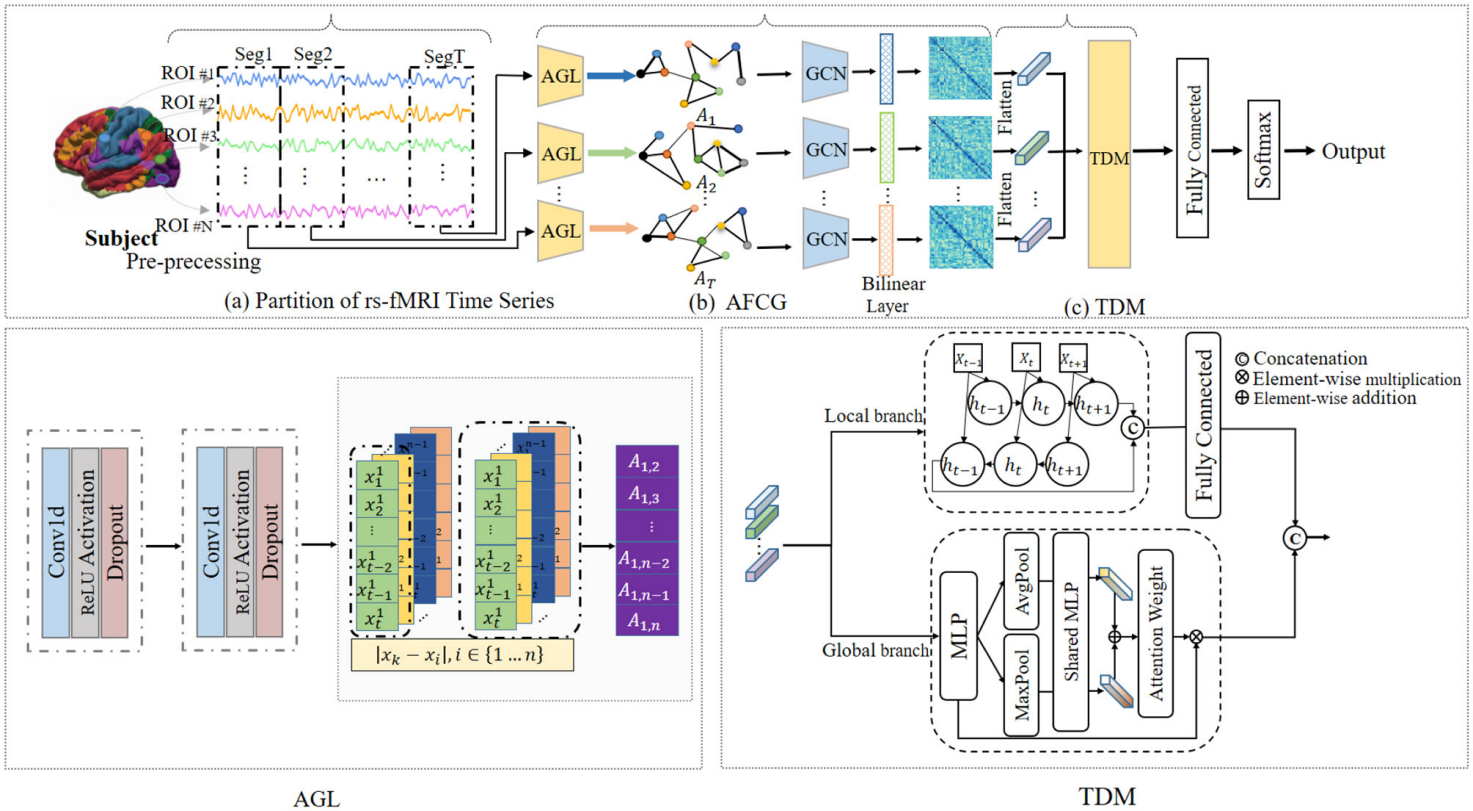
Overview of the proposed adaptive spatial-temporal neural network (ASTNet), including three components: **(A)** partitioning rs-fMRI time series into T segments via non-overlapping sliding windows, **(B)** adaptive functional connectivity generation (AFCG), where the adjacency matrix is first learned via adaptive graph learning (AGL) module for each time window and then fed into graph convolutional network (GCN), and **(C)** a temporal dependency mining module (TDM) to capture temporal dynamics across all time windows. With the output of the TDM, a fully-connected layer is further used for disease classification.

To solve this issue, we propose a novel adaptive spatial-temporal neural network (ASTNet) that can not only adaptively learn functional connectivities between brain ROIs but also mine global temporal dependencies in dFCs. As illustrated in Figure 1, the proposed ASTNet consists of three components, i.e., the partition of rs-fMRI time series, adaptive functional connectivity generation, and temporal dependency mining. Specifically, we first divide the rs-fMRI time series into multiple segments using non-overlapping sliding windows to characterize the temporal variability of fMRI time series. After that, for each time-series segment, we design an adaptive functional connectivity generation (AFCG) module that first adaptively learns FCs between ROIs and then use GCN to capture topological information of brain network. Finally, a temporal dependency mining (TDM) module which integrates local and global branches, is proposed to capture temporal dependencies from the spatially-dependent pattern sequences. Within the TDM module, the global branch investigates variations in individual points within the FC structure, such as the emergence or disappearance and the strengthening or weakening of connections, which is referred to as spatial variation. Meanwhile, the local branch examines temporal changes between dFCs, known as temporal variation. To further obtain subject-level representation, we concatenate the generated features from these two branches, followed by a fully connected layer for disease classification. Experimental results on 620 subjects in the ADHD-200 dataset demonstrate the effectiveness of our ASTNet in adaptive graph learning and temporal dependence mining. This demonstrates the importance and great potential of our model in ADHD identification, with great promise in practical applications.

## 2 Related work

### 2.1 Static FC-based method

Conventional FC-based methods usually first extract handcrafted features from functional connectivity networks and then train a classifier (e.g., support vector machine, SVM) for disease prediction (Bai et al., 2009; Jie et al., 2014a,b; Plis et al., 2014; Bi et al., 2018). For example, Bi et al. (2018) designed a random SVM cluster method for AD identification. This method firstly randomly selected samples and FC features to establish multiple SVMs, and then employed an ensemble strategy for the final prediction. Jie et al. (2014a) extracted and integrated multiple properties of static FC networks (e.g., connectivity strength and local clustering coefficient) for diagnosing brain diseases and achieved better performance compared with single network measures. Even so, these methods usually rely on handcrafted feature representations for classification models, thereby possibly producing sub-optimal classification performance.

More recently, deep learning methods have been proposed to automatically learn data-driven features from dFC networks. These methods offer a unified framework for fMRI feature learning and brain disorder prediction, ultimately achieving better performance. For example, Liang et al. (2021) proposed a novel convolutional neural network combined with a prototype learning (CNNPL) framework to classify brain functional networks for the diagnosis of autism spectrum disorder. Specifically, it used traditional convolutional neural networks to extract high-level features from pre-defined FCs and further designed a prototype learning strategy to automatically learn prototypes of each category for ASD classification. Eslami et al. (2019) proposed to extract the lower dimensional feature representation of FCs using an autoencoder, followed by a single layer perceptron (SLP) for ASD identification. Kawahara et al. (2017) developed three distinct convolutional layers—edge-to-edge (E2E), edge-to-node (E2N), and node-to-graph layer (N2G)—to capture the spatial characteristics of structural brain connectivity for cognitive and motor developmental score prediction in premature infants. Due to the graph-structured nature of brain functional networks, graph neural network (GNN), which can learn expressive graph representations, have shown significant potential in FC-based brain disease diagnosis. For example, Ktena et al. (2018) proposed learning a graph similarity metric using a siamese graph convolutional neural network for ASD classification. Yao et al. (2021) developed a mutual multi-scale triplet graph convolutional network for brain disorder diagnosis using functional or structural connectivity. Li et al. (2020) designed an ROI-aware graph convolutional layer that leveraged fMRI's topological and functional information for ASD diagnosis.

These deep learning methods greatly improve the efficiency and classification/regression performance in FC-based analysis due to their end-to-end architecture. However, these methods mainly study static patterns of brain networks, thereby ignoring the dynamic characteristics of brain FCs. Besides, GNN-based methods generally take a fixed graph structure as input, whose reliability remains to be discussed.

### 2.2 Dynamic FC-based method

Several dynamic functional analysis methods have recently been proposed for brain disease classification (Wang et al., 2019b, 2023; Yan et al., 2019; Gadgil et al., 2020; Lin et al., 2022; Liu et al., 2022; Liang et al., 2023). For example, Wang et al. (2019b) proposed a spatial-temporal convolutional-recurrent neural network (STNet) for Alzheimer's disease progression prediction using rs-fMRI time series. Specifically, a convolutional component was first employed to construct the FC within each time-series segment. Then, the long short-term memory (LSTM) units were used to model the temporal dynamics patterns of these successive FCs. Finally, a fully connected layer is used to perform disease progression prediction. Lin et al. (2022) developed a convolutional recurrent neural network (CRNN) for dynamic FCs analysis and automated brain disease diagnosis. In this method, a sequence of pre-constructed FC networks was input into three convolutional layers to extract temporal features, and an LSTM layer was used to capture temporal information from multiple time segments, followed by three fully connected layers for brain disease classification. To take advantage of spatio-temporal information of fMRI data, Yan et al. (2019) designed a multi-scale RNN framework to classify schizophrenia. Specifically, stacked convolution layers were used to extract different scale features, followed by a two-layer stacked Gated Recurrent Unit (GRU) to mine dynamic information conveyed in fMRI series. Gadgil et al. (2020) trained a spatio-temporal graph convolutional network (ST-GCN) on each segment of the BOLD time series to predict gender and age. In this method, a positive and symmetric “edge importance” matrix was first integrated to determine the importance of spatial graph edges. Then, three layers of ST-GC units were used to perform spatial graph convolution, followed by a fully connected layer for final prediction. Liang et al. (2023) proposed a self-supervised multi-task learning model for detecting AD progression, in which a masked map auto-encoder and temporal contrast learning were jointly pre-trained to capture the structural and evolutionary features of longitudinal brain networks. Liu et al. (2022) proposed a method based on nested residual convolutional denoising autoencoder (NRCDAE) and convolutional gated recurrent unit (GRU) for ADHD diagnosis. Specifically, the NRCDAE was used to reduce the spatial dimension of rs-fMRI and extract the 3D spatial features. Then, the 3D convolutional GRU was adopted to extract the spatial and temporal features simultaneously for classification. Although existing dynamic FC-based methods consider the temporal dynamics in the prediction of disease progression, those methods fail to capture the global temporal changing patterns of the whole brain (i.e., the longitudinal network-level patterns).

## 3 Materials and method

In this section, we introduce the materials used in this work, the proposed method, as well as implementation details.

### 3.1 Material

#### 3.1.1 Data acquisition

We use the ADHD-200 dataset to validate the effectiveness of the proposed method. The ADHD-200 dataset includes 973 subjects collected from eight different imaging sites. Specifically, the dataset contains 362 ADHD patients, 585 normal controls (NC), and 26 undiagnosed subjects and can be accessed from the NeuroImaging Tools & Resource Collaboratory (NITRC) website.^1^ Each participant's data in the ADHD-200 dataset consists of a resting-state functional MRI scan, a structural MRI scan, and the corresponding phenotypic information. Note that ADHD patients in the ADHD-200 dataset are further categorized into three subtypes: ADHD-Combined, ADHD-Hyperactive/Impulsive, and ADHD-Inattentive. To simplify the binary classification task, all subtypes in the ADHD-200 dataset are uniformly labeled as 1. During the ADHD-200 Global Competition, the ADHD-200 dataset is divided into a training set and a test set, each with corresponding phenotypic information. The numbers of subjects are 768 and 197, respectively. In this paper, we also follow this division in our experiments for a fair comparison. Note that in our performance evaluation, we exclude 26 subjects without released labels in the test set. Furthermore, we also discard subjects from the Pitt and Washu imaging sites in our study because their training sets only contained normal control (NC) subjects. Thus, a total of 620 subjects are used in this study, including 340 ADHD patients and 280 NCs. The detailed demographic information of involved subjects and data partition for experiments are provided in Table 1.

**Table 1:** Demographic information and data partition of the studied subjects from ADHD-200 dataset.

**Item**	**PKU**	**NYU**	**OHSU**	**NI**	**KKI**	**Total**
**Training dataset**						
Number	194	216	79	48	83	620
NC	116	98	42	23	61	340
ADHD	78	118	37	25	22	280
Gender (M/F)	144/50	140/76	43/36	31/17	46/37	404/216
**Test dataset**						
Number	51	41	34	25	11	162
NC	27	12	28	14	8	89
ADHD	24	29	6	11	3	73
Gender (M/F)	32/19	28/13	17/17	12/13	10/1	99/63

M, male; F, female.

#### 3.1.2 Data pre-processing

All resting-state fMRI data used in our study were preprocessed by the C-PAC pipeline.^2^ This pipeline includes several processing steps such as skull stripping, slice timing correction, head motion realignment, intensity normalization, band-pass filtering (0.01–0.1 Hz), and the regression of white matter, cerebrospinal fluid, and motion parameters. To minimize the impact of head motion on our results, we first removed fMRI data from participants whose heads moved more than 2.0 mm in any direction or 2° in any rotation. After that, we performed structural skull stripping and then mapped the remaining fMRI data to the Montreal Neurological Institute (MNI) space. A 6mm Gaussian kernel was used to spatially smooth the rs-fMRI data. Note that further our analysis excluded subjects with a frame displacement exceeding 2.5 min (FD > 0.5). Finally, the automated anatomical labeling (AAL) template was used to extract the mean rs-fMRI time series for a set of 116 pre-defined ROIs.

### 3.2 Method

As illustrated in Figure 1, the proposed ASTNet includes *(1)* partition of rs-fMRI time series, *(2)* adaptive functional connectivity generation, and *(3)* temporal dependency mining.

#### 3.2.1 Partition of rs-fMRI time series

To characterize the temporal variability of fMRI series, we first employ the sliding window strategy to partition all rs-fMRI time series into *T* non-overlapping windows, each with a fixed window size *L*. Specifically, for each window, we represent the segmented time series as $G_{t}={\left(v_{1}^{t},v_{2}^{t},...,v_{N}^{t}\right)}^{⊤}\in R^{N\times L}\left(t=1,\cdots \;,T\right)$, where the *t* represents the *t*-th segments and *N* denotes the number of nodes. In our paper, the window size *L* is set as 20. For PKU, NYU, OHSU, NI, and KKI sites, the lengths of extracted fMRI time series are 231, 171, 72, 256, and 119 and the corresponding TR is 2.5, 1.96, 2, 2.5, and 2s, respectively. Since each site has a different scanning time (*i.e*., length of fMRI time series), we obtain *T* = 5, *T* = 12, *T* = 8, *T* = 3, and *T* = 11 segments for these five sites, respectively. The reason for choosing such window length is that window sizes around 30–60 s can provide a robust estimation of the dynamic fluctuations in rs-fMRI data (Wang et al., 2021). For each subject, the time-series segment S will be considered as the input of the proposed network.

#### 3.2.2 Adaptive functional connectivity generation

In order to better explore the spatial relationships between brain regions, we employ an adaptive graph learning (AGL) strategy to learn functional connectivities, instead of relying on prior knowledge or manual labor. We define a non-negative function Equation (1) with a learnable weight vector $\omega ={\left(\omega_{1},\omega_{2},...,\omega_{F_{d_{e}}}\right)}^{T}\in R^{F_{de}*1}$ based on the graph data *G*_*t*_ for each window to represent the connection between any two brain nodes *x*_*m*_ and *x*_*n*_:


(1) $$\begin{array}{l} A_{mn}=g\left(x_{m},x_{n}\right)=\frac{exp\left(RELU\left(\omega^{T}|x_{m}-x_{n}|\right)\right)}{\overset{N}{\underset{n=1}{\sum }}exp\left(RELU\left(\omega^{T}|x_{m}-x_{n}|\right)\right)}, \end{array}$$

where *x*_*i*_ represents the fMRI data of the *i*-th brain ROI, nonlinear activation function ReLU guarantees *A*_*mn*_ is nonnegative, and the softmax operation normalizes each row of *A*. To introduce prior knowledge, we incorporate the following regularization loss Equation (2):


(2) $$L_{graph\_learning}=\sum_{m,n=1}^{N}||x_{m}-x_{n}|{|}_{2}^{2}A_{mn}+\lambda ||A|{|}_{F}^{2}.$$

That is, the smaller distance ||*x*_*m*_−*x*_*n*_||_2_ between *x*_*m*_ and *x*_*n*_, the larger *A*_*mn*_ is. This regularization allows nodes/ROIs with similar features to have greater connection weights. Furthermore, considering the sparsity nature of brain functional network (*i.e*., brain graph), the second term is introduced, where λ≧0 is a regularization parameter. Through the proposed graph learning mechanism, we obtain an adaptively learned adjacency matrix *A* used for the subsequent graph convolution operation Equation (3):


(3) $$\begin{array}{l} H^{l+1}=\sigma \left(AH^{l}W^{l}\right), \end{array}$$

where *H*^*l*^ is the time series signal characteristics of brain network nodes in layer *l*, *A* represents the learned adjacency matrix, *W* denotes a learnable weight matrix, and σ is the activation function. The fundamental principle underlying graph convolution is the iterative aggregation of neighboring node information to update the feature representation of the central node. Finally, we get the functional connectivity matrix by Equation (4):


(4) $$\begin{array}{l} S_{t}=H^{l}{\left(H^{l}\right)}^{T}, \end{array}$$

where *H*^*l*^ denotes the final node features generated from GCN and *S*_*t*_ measures the degree of second-order dependency between ROIs.

#### 3.2.3 Temporal dependency mining

To capture temporal dynamic information within fMRI series across temporal dimension, we design the temporal dependency mining (TDM) module which includes two parallel architectures, i.e., local and global branches. The local branch is designed to explore the temporal evolution of adjacent sliding windows, providing insights into fine-grained changes. The global branch is used to capture the evolutionary patterns across all timestamps. Details are introduced below.

##### 3.2.3.1 Local temporal dependency mining branch

To capture the local temporal dependency of dFCs, we propose the use of a bi-directional Gated Recurrent Unit (BiGRU), which is a type of recurrent neural network (RNN). Different from unidirectional GRU, the bidirectional GRU (BiGRU) consists of two GRUs, where one GRU scans the sequence from the beginning to the end, while the other scans the sequence from the end to the beginning. This bidirectional structure enables the model to consider both past and future information simultaneously, thereby enhancing the accuracy of feature information capture in sequential data. Mathematically, the BiGRU can be represented as Equation (5):


(5) $$\begin{array}{l} y_{t}=GRU\left(x_{t},\vec{h_{t-1}}\right)⊕GRU\left(x_{t},\overset{⃖}{h_{t-1}}\right), \end{array}$$

where *x*_*t*_ denotes initial temporal state, *h*_*t*_ represents hidden state, and arrows represent different operation directions. The GRU is composed of two gating mechanisms, including the reset gate and the update gate. The calculation formulas for the GRU unit are as follows:


(6) $$\begin{array}{r} z_{t}=\sigma \left(W_{z}\cdot \left[h_{t-1},x_{t}\right]\right),\\ r_{t}=\sigma \left(W_{r}\cdot \left[h_{t-1},x_{t}\right]\right),\\ {\tilde{h}}_{t}=tanh\left(W\cdot \left[r_{t}*h_{t-1},x_{t}\right]\right),\\ h_{t}=\left(1-z_{t}\right)*h_{t-1}+z_{t}*{\tilde{h}}_{t}. \end{array}$$

In Equation (6), the reset gate operation *r*_*t*_ controls the fusion of new input information with the previous “memory,” and the update gate *z*_*t*_ influences the amount of information to be forgotten from the previous moment. In the second formula, *W*_*r*_ denotes a weight matrix, while *r*_*t*_ is obtained by linearly transforming the concatenated matrix of *x*_*t*_ and *h*_*t*−1_. This value is subsequently utilized in the third formula to update the hidden information of the candidate. For ease of understanding:


(7) $$\begin{array}{l} {\tilde{h}}_{t}=tanh\left(x_{t}W_{xh}+\left(r_{t}⊙h_{t-1}\right)W_{hh}+b_{h}\right) \end{array}$$

In Equation (7) the value of *r*_*t*_ in the update gate influences the amount of information to be forgotten from the previous moment, as indicated by the Hadamard product with *h*_*t*−1_. The first equation represents the update gate, while the fourth equation controls the extent to which previous information is incorporated into the current state. In the fourth equation, the closer *z*_*t*_ is to 1, the more information it retains or “remembers.” (1−*z*_*t*_)*h_*t*−1_ selectively forgets parts of the previous hidden state, while $z_{t}*{\tilde{h}}_{t}$ selectively incorporates candidate hidden states. In summary, the fourth equation combines forgetting some information passed down from *h*_*t*−1_ with incorporating relevant information from the current node, resulting in the final memory representation *h*_*t*_. In this way, we can obtain the final local time dependency information between adjacent time-sliced fMRI data by recursively transmitting hidden state information.

##### 3.2.3.2 Global temporal dependency mining branch

We employ global attention to capture the global temporal dependency of dFCs. For each segment, we first employ an MLP to extract potential hidden abnormal connection information, ensuring that spatial information at different stages remains intact despite the temporal interactions. Specifically, the upper triangular data of the symmetric matrix is first converted to a one-dimensional vector *x* and then fed into the MLP to obtain the characteristic information *F*_*t*_, represented as Equation (8):


(8) $$\begin{array}{l} F_{t}=g\left(\overset{M}{\underset{i=0}{\sum }}w_{i}x_{i}\right), \end{array}$$

where *w*_*i*_ is learnable weight and *g* is activation function. Then, we incorporate a global attention mechanism to capture temporal dependencies between dynamic FCs. The formula of global attention is defined as Equation (9):


(9) $$\begin{array}{l} M_{c}\left(F\right)=\sigma \left(MLP\left(AvgPool\left(F\right)\right)+MLP\left(MaxPool\left(F\right)\right)\right), \end{array}$$

where *F* represents the function connectivity information processed by previous MLP on T segments and σ denotes the sigmoid function. Note that the MLP weights are shared for both inputs and the ReLU activation function. The obtained attention weights (i.e., *M*_*c*_(*F*)) are used to combine information from multiple windows, resulting in a final feature representation expressed as a one-dimensional vector denoted as $\bar{d}$. Finally, we concatenate the output of global and local branches, yielding a one-dimensional vector. Then, this one-dimensional vector is fed into three fully connected layers to obtain the final classification result.

It's worth noting that, to avoid the trivial solution (i.e., ω = (0, 0,…, 0), which is due to minimizing the above loss function *L*_*graph*_*learning*_ independently, we utilize it as a regularized term to form the final loss function Equation (10):


(10) $$\begin{array}{l} L_{loss}=L_{cross\text{\_}entropy}+L_{graph\text{\_}learning}, \end{array}$$

where *L*_*cross*_*entropy*_ denotes the categorical_crossentropy of the classification task.

### 3.3 Implementation

We implement the framework using Python 3.7 and Pytorch library. For each subject, the adjacency matrix is constructed via our designed AGL strategy, where a random initialization technique initializes the vector ω according to a normal distribution. Subsequently, the graph convolution process comprises three GCN layers, followed by batch normalization, ReLU activation, and a dropout rate of 0.5. We then perform a dot product operation on the representation generated from the GCN layers to construct symmetric matrices describing the degree of correlation between nodes. To reduce dimensionality, we flatten the upper triangular portion of each matrix into a vector. This vector is subsequently fed into an MLP consisting of three fully connected layers. Additionally, we incorporate two dropout layers to mitigate overfitting.

Subsequently, we employ an attention mechanism to obtain attention weights and compute the weighted sum of the vector data. This process yields a 32-dimensional vector, which serves as the final output of the global branch. For local analysis using the BiGRU, we perform experiments with different numbers of units, specifically 4, 16, and 64, for training data from various sites.

## 4 Experiment and result analysis

### 4.1 Methods for comparison

In the experiments, we compare our ASTNet model with the following eight methods, including the baseline methods and any other variants of the proposed method.

1. MLP (Tolstikhin et al., 2021): in this method, the static FC matrix for each subject is directly used as the input of the MLP model. Specifically, the MLP model comprises three fully-connected layers with hidden neurons of 1,024, 256, and 32, respectively.
2. AE (Wang et al., 2014): auto-encoder (AE) is an unsupervised learning model that can learn a mapping supervised by input *X* itself. Specifically, AE extracts useful features from brain networks through bottleneck-like fully connected layers. The hidden layer dimension is determined by the data length in different sites.
3. GCN (Kipf and Welling, 2016): this method first uses two layers of GCN based on Pearson correlation to update spatial correlation between ROIs where the data length in different sites determines dimensions. Then, the FCs, calculated from the dot product of node features, are used as the input to construct a three-layer MLP model with hidden neuron number of 1,024, 256, and 32, respectively.
4. AGL_s: in this method, FCs are learned by the adaptive method. Static adaptive graph learning (AGL_s) method replaces the Pearson correlation matrices with adaptive brain networks as the input. The network structure is the same as the previous MLP method.
5. BiGRU (Chung et al., 2014): This method partitions fMRI data with a constant length of 20 time points. For each segment, we build a functional connectivity matrix. Then, these matrices are sent into the BiGRU model to study their temporal change information with different numbers of units, specifically 4, 16, and 64, for training data.
6. AGL_d: dynamic adaptive graph learning method (AGL_d) implies adaptive learning to construct adjacency matrices on each segment. Then, the TDM module is used to analyze time-varying information between different sliding window data. Specifically, the local branch adopts a two layers BiGRU with 4 units to make the final classification, and the Global branch explores temporal and spatial variability using global attention where MLP has three hidden layers 1,024, 256, and 32, respectively, and the ratio is set as 3 in attention mechanism.

Note that, we both have static and dynamic experiments for method MLP and GCN, named as MLP_s, MLP_d, GCN_s, and GCN_d. And we apply global branch in the TDM to mine temporal dependency for MLP_d and adopt the whole TDM module for GCN_d classification.

### 4.2 Experiment settings

We evaluate the proposed method on five different sites (i.e., PKU, NYU, OHSU, NI, and KKI) of the ADHD database based on rs-fMRI data. We divide data on each site into training data and test data, following Global Competition. The test set is unseen during the training stage.

To evaluate classification performance, three metrics are used, including accuracy (ACC), sensitivity (SEN), and specificity (SPE). These metrics are defined as follows: ACC = (TP + TN)/(TP + TN + FP + FN), SEN = TP/(TP + FN), SPE = TN/(TN + FP). Here, TP, TN, FP, and FN represent true positive, true negative, false positive, and false negative values, respectively. Higher values for these metrics indicate better classification performance.

### 4.3 Classification performance

The quantitative results achieved by different methods in the binary classification tasks are reported in Table 2. From Table 2, one could have three main observations.

**Table 2:** Accuracy (ACC) values achieved by our proposed ASTNet and eight competing methods on five sites (*i.e*., PKU, NYU, OHSU, NI, and KKI) of ADHD-200 dataset.

**Method**	**PKU**			**NYU**			**OHSU**			**NI**			**KKI**			**Avg-ACC**
	**ACC**	**SEN**	**SPE**	**ACC**	**SEN**	**SPE**	**ACC**	**SEN**	**SPE**	**ACC**	**SEN**	**SPE**	**ACC**	**SEN**	**SPE**	
MLP_s	70.6	85.2	54.2	48.8	58.3	44.8	67.6	75.0	33.3	64.0	78.6	45.5	81.8	100.0	33.3	66.6
AE	66.7	88.9	41.7	53.7	66.7	48.3	70.6	82.1	16.7	64.0	78.6	45.5	72.7	75.0	66.7	65.5
GCN_s	64.7	66.7	62.5	61.0	75.0	55.2	70.6	78.6	33.3	68	71.4	63.6	72.7	100.0	0.0	67.4
AGL_s	68.6	74.1	62.5	70.7	58.3	75.9	82.4	**96.4**	16.7	60.0	78.6	36.4	81.8	100.0	33.3	72.7
BiGRU	62.7	74.1	50.0	53.7	58.3	51.7	64.7	71.4	33.3	52.0	42.9	**63.6**	72.7	87.5	33.3	61.2
MLP_d	70.6	88.9	50.0	48.8	66.7	41.4	73.5	82.1	33.3	56.0	71.4	36.4	72.7	87.5	33.3	64.3
GCN_d	64.7	74.1	54.2	70.7	66.7	72.4	73.5	85.7	16.7	68.0	92.9	36.4	72.7	100.0	0.0	69.9
AGL_d	70.6	81.5	58.3	70.7	58.3	75.9	76.5	89.3	16.7	68.0	85.7	45.5	81.8	87.5	66.7	73.5
ASTNet (ours)	**74.5**	**85.1**	**62.5**	**75.6**	**75.0**	**75.9**	**85.3**	89.3	**66.7**	**76.0**	**92.9**	54.5	**90.9**	**100.0**	**66.7**	**80.5**

Avg-ACC, the average ACC across these five sites (%).

Best results are shown in bold.

First, our proposed method and its variants (*i.e*., AGL_d and GCN_d) generally achieve better performance compared with the baseline methods (*i.e*., MLP, BiGRU, and Auto-Encoder) in the classification task. For example, in terms of ACC values, ASTNet achieved an improvement of 13.9%, compared with the best baseline method (with 66.6%) in ADHD classification. This demonstrates that our designed adaptive functional connectivity learning strategy and temporal dependency mining module can help extract more discriminative fMRI features, thus enhancing classification performance. Second, our proposed ASTNet and its variants outperform those methods without considering the temporal dynamics (e.g., with GCN_s and AGL_s) in terms of most metrics. In particular, the SEN values produced by our ASTNet in site PKU and NYU are 85.1 and 75.0%, which is higher than other methods. These results suggest that our TDM module can effectively capture dynamic changes in rs-fMRI time series. Finally, our ASTNet is superior to its variants (*i.e*., AGL_d and *GCN*_*d*). This result implies that the adaptive functional connectivity learning strategy and TDM module help boost the learning performance of ASTNet.

### 4.4 Interpretable analysis of the learned FCs

**Figure 2:**
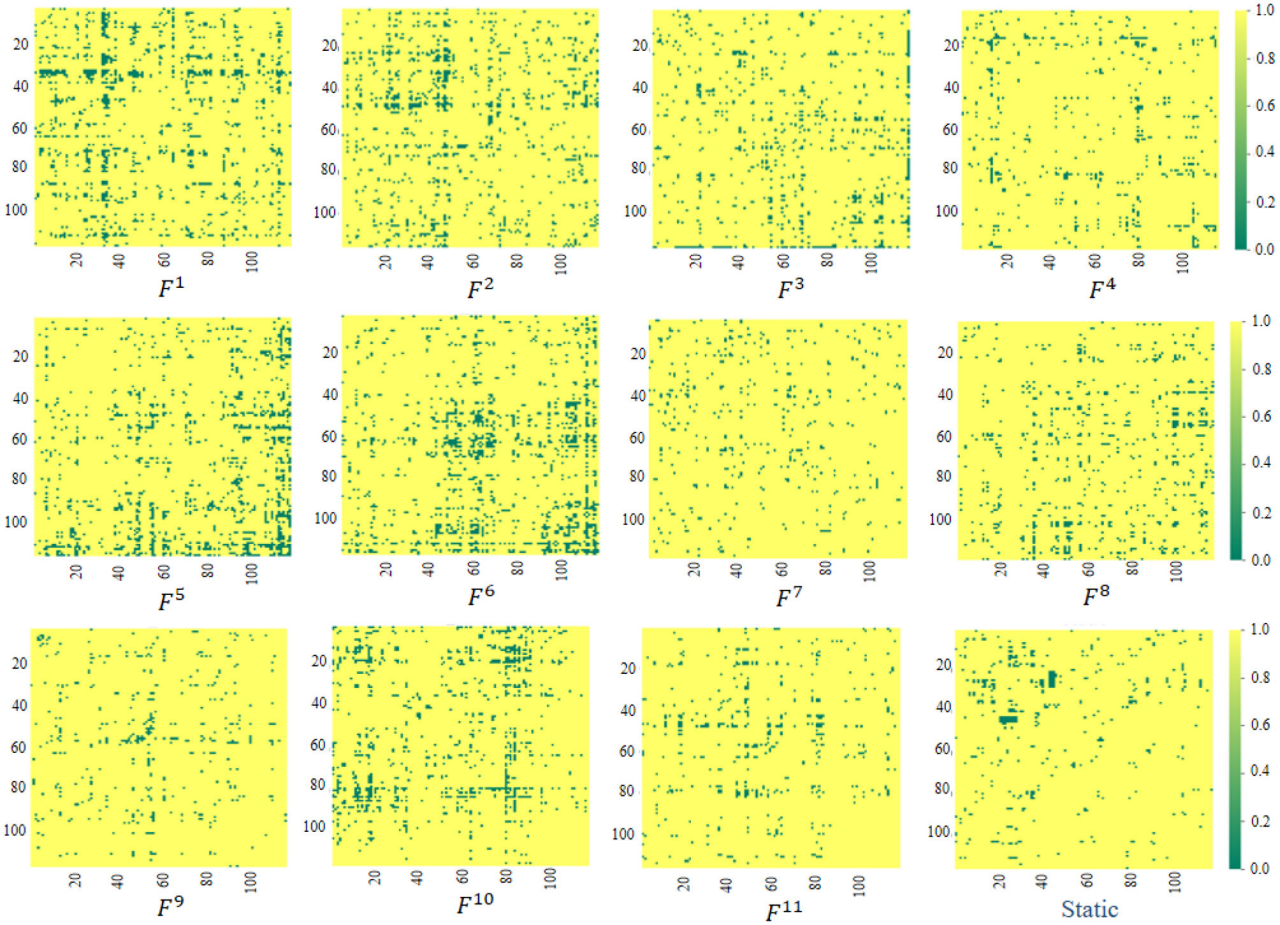
Visualization of group difference matrices generated by our learned dynamic FC networks and static network. Note that *p*-values > 0.05 are set to 1 (shown in yellow), while those ≤0.05 were set to 0 (shown in green). The *F*^*i*^(*i* = 1, …, 11) repesents the group difference matrices generated by the *i*-th segment.

The proposed ASTNet can automatically learn dFCs in a data-driven manner, which differs from previous studies that rely on predefined FC networks (e.g., via Pearson's correlation). We now further analyze the FC networks learned by the proposed adaptive method. Specifically, as introduced in Section 3.2.2, the AFCG can learn new connectivity strength between each central node/ROI and all the remaining N-1 ROIs. Therefore, we can generate a fully connected FC network based on the learned connectivity vector. Given the size of the sliding window, different lengths of data will result in different numbers of segments. Taking PKU site as an example, we can construct *K* = 11 dynamic FC networks for each subject, with each network corresponding to a segment. Finally, using the standard t-test, we measure the group difference between ADHD and NC via p-values, with group difference matrices visualized in Figure 2. For comparison, in Figure 2, we also report the group difference of the stationary FC network that is constructed via measuring Pearson correlation coefficients between fMRI time series of pairwise brain ROIs. Note that the obtained *p*-values were binarized (i.e., setting *p*-values more than 0.05 to 1 and 0 otherwise) for clarity in Figure 2. From Figure 2, we have the following observations.

*First*, from *F*^*i*^, *i* = 1, …, 11 in Figure 2, we can observe that the group difference matrices generated by different segments exhibit significant differences, which further validates the temporal variability of brain networks. *Second*, by comparing our learned *F*^*i*^ and Static in Figure 2, it can be found that the dynamic FC network learned by our ASTNet shows superiority over the pre-defined static network in identifying disease-related functional connectivities and ROIs. For example, Several ROIs, such as the anterior cingulate and paracingulate gyri node (ACG.L) in *F*^1^, the superior parietal gyrus node (SPG.L) in *F*^6^, and the cerebellum nodes in *F*^5^, are detected by our dynamic FC networks in AD vs. NC classification. These findings aligns with previous AD-related studies, which further demonstrates the learned FCs by our ASTNet have good interpretability.

### 4.5 Comparison with state-of-the-art methods

We further compare the proposed ASTNet with seven state-of-the-art (SOTA) methods designed for ADHD analysis, including PCA-LDA (Dey et al., 2012), EM-MI (Dou et al., 2020), 3D CNN (Zou et al., 2017), SASNI (Zhang et al., 2017), SPAE (Cao et al., 2023), STAAE (Dong et al., 2020), and KD-Transformer (Zhang et al., 2022). Note that all the methods use the standard training/test sets division by the data set. The classification results achieved by different methods are reported in Table 3, with the best results highlighted in bold. From Table 3, we can have the following findings.

**Table 3:** Quoted results from literature on ADHD-200 dataset.

**Method**	**PKU**	**NYU**	**OHSU**	**NI**	**KKI**	**Avg-ACC**
PCA-LDA (Dey et al., 2012)	62.7	70.7	73.5	72.0	72.7	70.3
EM-MI (Dou et al., 2020)	70.6	63.4	–	–	81.8	70.4
3D CNN (Zou et al., 2017)	63.0	70.5	–	–	72.8	66.0
SASNI (Zhang et al., 2017)	74.5	70.7	79.4	72.0	63.6	72.0
SPAE (Cao et al., 2023)	70.6	65.4	65.9	76.0	73.6	70.5
STAAE (Dong et al., 2020)	79.5	82.2	75.4	63.7	76.6	75.5
KD-transformer (Zhang et al., 2022)	70.6	**82.9**	85.3	72.0	90.9	80.3
ASTNet (ours)	**74.5**	75.6	**85.3**	**76.0**	**90.9**	**80.5**

Best results are shown in bold (%).

*First*, the proposed ASTNet outperforms seven SOTA methods in ADHD classification task on the ADHD-200 dataset, which implies that our ASTNet can learn more discriminative features for ADHD identification. *Second*, Compared with static methods (*i.e*., PCA-LDA, 3D CNN, SASNI, and KD-Transformer), the methods (*i.e*., SPAE, STAAE, and KD-Transformer) that consider temporal dynamics in fMRI series achieves relatively better performance. This suggests that temporal information conveyed in fMRI series plays an important role in distinguishing ADHD patients from normal controls.

*Third*, the ACC of our ASTNet achieves an improvement of 5% compared with the STAAE that designs a spatiotemporal attention auto-encoder long-distance dependency in time. This finding further demonstrates the superiority of our ASTNet in dynamic brain network learning and brain disorder classification.

## 5 Discussion

In this section, we explore the influence of different sliding window sizes, compare the proposed method with its degraded variants, and discuss several limitations of the current work and future work.

### 5.1 Influence of sliding window size

**Figure 3:**
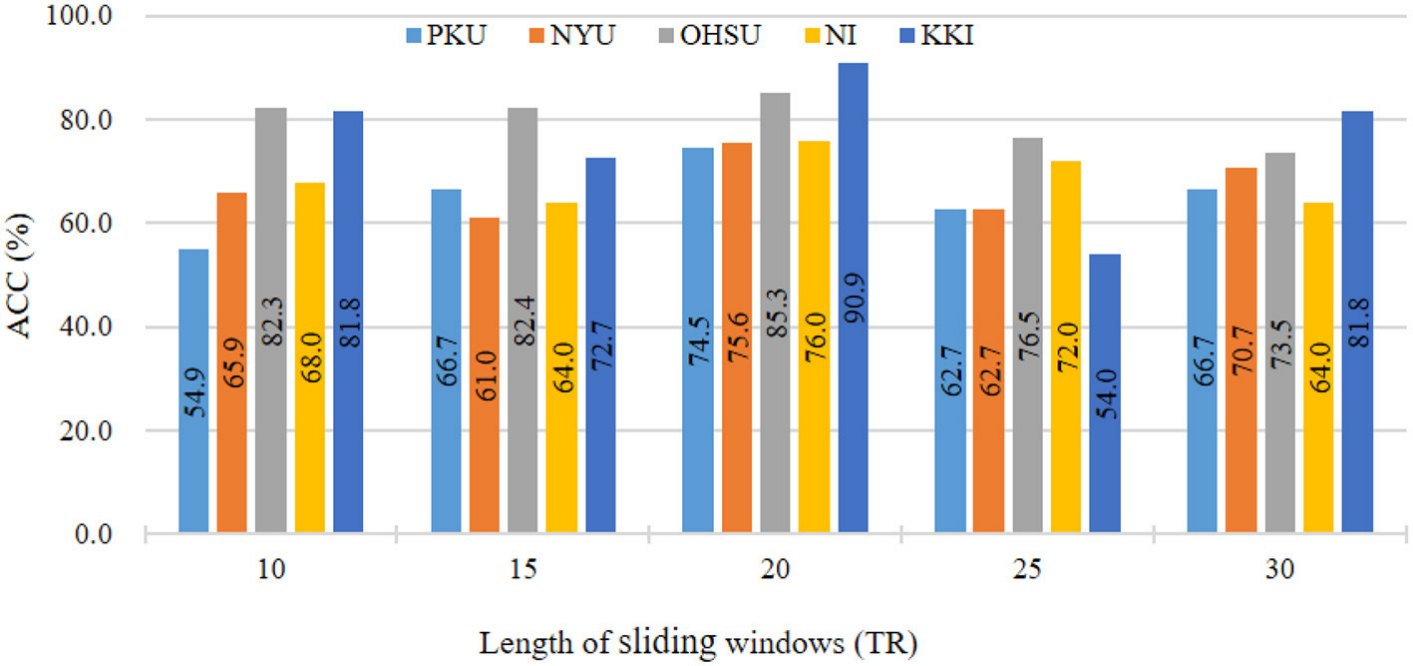
Results of the proposed ASTNet method with respect to different sliding windows length in ADHD vs. NC on different sites.

In main experiments, we divide fMRI series using sliding window strategy with window size of 20. To investigate the influence of different sliding window sizes on results, we vary the values of sliding window size within [10, 15, ⋯ , 30]. The results in ADHD classification on five sites are reported in Figure 3. As shown in Figure 3, we can see that our the classification accuracy of our ASTNet fluctuates to a certain extent as the window size increases. When window size is 20, our method achieves its peak performance across different sites, which validates that our selected window size is reasonable.

### 5.2 Ablation study

To demonstrate the effectiveness of each module in the proposed ASTNet, we further compare our ASTNet with its degenerated variants, including (1) GCN_d without incorporating adaptive graph learning (AGL) module, (2) AGL_d without incorporating GCN module, (3) ASTNet_G without global branch and (4) ASTNet_L without local branch. The experiment results of our ASTNet and its variants are reported in Table 4.

**Table 4:** Ablation results on ADHD-200 dataset.

**Method**	**PKU**			**NYU**			**OHSU**			**NI**			**KKI**			**Avg-ACC**
	**ACC**	**SEN**	**SPE**	**ACC**	**SEN**	**SPE**	**ACC**	**SEN**	**SPE**	**ACC**	**SEN**	**SPE**	**ACC**	**SEN**	**SPE**	
GCN_d	64.7	74.1	54.2	70.7	66.7	72.4	73.5	85.7	16.7	68	92.9	36.4	72.7	100.0	0.0	69.9
AGL_d	70.6	81.5	58.3	70.7	58.3	75.9	76.5	89.3	16.7	68.0	85.7	45.5	81.8	87.5	66.7	73.5
ASTNet_G	72.5	81.5	62.5	70.7	50.0	79.3	82.4	**100.0**	0.0	72.0	92.9	45.5	90.9	87.5	**100.0**	77.7
ASTNet_L	62.7	74.1	50.0	68.3	41.7	**79.3**	64.7	67.9	50.0	**80.0**	92.9	**63.6**	81.8	87.5	66.7	71.5
ASTNet (ours)	**74.5**	**85.1**	**62.5**	**75.6**	**75.0**	75.9	**85.3**	89.3	**66.7**	76	**92.9**	54.5	**90.9**	**100.0**	66.7	**80.5**

Best results are shown in bold (%).

It can be found from that our ASTNet consistently outperforms GCN_d that fails to adaptively learn FC strength. This implies that our designed adaptive graph learning strategy can automatically generate more reliable FC network for subsequent analysis, thus boosting model performance. In addition, we can observe that our ASTNet is superior to ASTNet_G without modeling long-term dependencies among dynamic functional connectivities (dFCs). Besides, our ASTNet achieves better performance than ASTNet_L that can not capture local temporal dependency in dFCs. These observations further demonstrate the advantage of our ASTNet, which simultaneously uses global and local branches in fMRI temporal feature learning.

### 5.3 Limitations and future work

While our work achieves good results in automatically identifying ADHD using fMRI data, several issues still need to be considered in the future to further improve the performance of the proposed method. First, considering the small-sample-size issue of fMRI data, we will employ transfer learning and pretraining strategies to further enhance model generalization. Second, different brain image modalities, such as structural MRI and Positron Emission Tomography (PET), can provide complementary information for ADHD diagnosis. Integrating multimodal neuroimages would be an interesting avenue to pursue, which will be our future work. Finally, we only construct the functional connectivity matrix based on the AAL atlas with 116 pre-defined ROIs in this work. In the future, we will explore multi-scale functional connectivity networks divided by multiple brain atlas to capture complementary topological information.

## 6 Conclusion

In this paper, we propose an end-to-end adaptive spatial-temporal neural network for ADHD classification using rs-fMRI time-series data. Specifically, we first divide fMRI data into non-overlapping segments to characterize the temporal variability. Then, a adaptive functional connectivity generation (AFCG) module is used model spatial dependencies between brain ROIs for each segment. In particular, within the AFCG, a adaptive graph learning strategy is designed to learn functional connectivity strength a data-driven manner. Finally, we develop a temporal dependency mining (TDM) module that integrates global and local branches to capture the temporal dynamics across multiple time segments. Extensive experiments on the dataset demonstrate the superiority of our ASTNet over several state-of-the-art methods, demonstrating its potential in identifying ADHD.

## Data availability statement

The original contributions presented in the study are included in the article/supplementary material, further inquiries can be directed to the corresponding author.

## Author contributions

BQ: Writing – original draft. QW: Writing – review & editing. XL: Validation, Writing – original draft. WL: Writing – original draft. WS: Writing – review & editing. MW: Writing – original draft, Writing – review & editing.

## Footnotes

^1^ https://fcon 1000.projects.nitrc.org/indi/adhd200/

^2^ http://preprocessed-connectomes-project.org/abide/
